# A flexible way to study composites in ecology using structural equation modeling

**DOI:** 10.1038/s41598-025-88675-0

**Published:** 2025-02-07

**Authors:** Xi Yu, Florian Schuberth, Jörg Henseler

**Affiliations:** 1 Faculty of Engineering Technology, University of Twente, P.O. Box 217, 7500 AE Enschede, The Netherlands; 2 Nova Information Management School, Universidade Nova de Lisboa, Campus de Campolide, 1070-312 Lisbon, Portugal

**Keywords:** Plant ecology, Animal ecology, Henseler–Ogasawara specification, Collective effects, Emergent variable, Plant ecology, Ecology, Ecology, Climate-change ecology, Ecological modelling

## Abstract

Composites, which refer to weighted linear combinations of variables, are receiving increasing attention in the field of ecology. In practice, however, researchers relying on the common approaches to study composites encounter limitations in flexibly specifying composites with structural equation modeling (SEM). To enrich the researchers’ statistical toolbox and to flexibly model composites in structural equation models, we introduce the Henseler–Ogasawara (H–O) specification to the field of ecology. As we show in this paper, this approach can not only mimic the common approaches such as the one-step and two-step approaches, but also offers improvements. Compared to the two-step approach, the H–O specification explicitly models composites, i.e., it takes into account the formation of the composites and it allows modeling composites with free weights and with fixed weights, i.e., unknown-weight and fixed weight composites. Consequently, this specification allows for a more in-depth model assessment. Compared to the one-step approach, the H–O specification offers more modeling flexibility. For example, it allows researchers to specify the effects of other variables on a composite. Consequently, conceptual models can be more adequately represented by the statistical model using this specification. To demonstrate these advantages, we provide an ecological illustrative example including the R code to reproduce our results. Specifically, we present different H–O specifications and compare them statistically. Our analysis also shows that the specified model, closest to the conceptual model of the illustrative example does not adequately describe the data. Instead, a model that does not assume that all covariances between the components of the composites are accounted for by the composites fits the data well.

## Introduction

Structural equation modeling (SEM) is a flexible modeling framework that has been widely used not only by researchers in ecology (e.g.,^1–5^), but also in various other research fields such as organizational research^6^, psychological research^7^, clinical research^8^, and information systems research^9^. Its flexibility is underscored by the fact that a wide range of techniques can be considered as special cases of SEM^10^, such as regression analysis, analysis of variance, and confirmatory factor analysis. Arguably, one of the greatest advantages of SEM is its ability to empirically validate a model through an overall model fit assessment, in which the sample variance-covariance matrix is compared with the model-implied counterpart to assess the validity of the model^11^.

Researchers using SEM may encounter three different types of variables, namely (i) observed variables, (ii) latent variables, and (iii) composites^12–14^. An *observed variable* is a variable whose structure is fully epistemically accessible, i.e., the variable structure can be inferred with certainty from the data^15^. An ecological example of an observed variable is the height of a tree measured in meters, e.g.,^16^. In this case, we assume that the height of a tree can be inferred with certainty from the data. For example, if the measured height of a tree is 5 meters, we assume that it is certain that the tree is 5 meters tall or at least that any imprecision is negligible. In contrast, the structure of a *latent variable* cannot be inferred with certainty from the data, i.e., inferring the structure of the variable from the structure of the data is error-prone. Therefore, to study latent variables, observed variables are used as measures, also called *effect indicators*^17^. An ecological example of a latent variable is the “overall size” of blackbirds^18^. The overall size is measured by four morphological traits, i.e., tarsus, phalanx, wing, and tail length. A certain length of the tarsus does not allow us to conclude with certainty the overall size of a blackbird. In addition to observed and latent variables, ecologists also study *composites*. Composites are variables constructed from other variables, called *components*, usually as (weighted) linear combinations. Composites “represent the influences of collections of other variables” [^14^, p. 143]. For example, the composite “soil mineral” was used to study the collective effect of zinc (Zn), iron (Fe), and phosphorus (P) concentrations on the ratio of nirS and nirK^19^. Another example from animal ecology is the composite “predation risk” (to which herbivores are exposed), which has been used to study the collective effects of woody cover, distance to drainage, and landscape curvature on a species’ recurrence index^20^. Similarly, a composite can be used to study the collective effects on its components, i.e., the composite is affected by other variables in the model^21^. For example, composites have been used for contemporary nutrients, energy supply, biodiversity, chemodiversity and molecular traits to study the effects of climate change and human impacts on these composites^22^.

Despite the importance of composites in the field of ecology, the approaches commonly used to study composites in SEM unnecessarily limit the researcher compared to those who study only observed and latent variables. To study composites in SEM, ecological researchers use either the two-step approach or the one-step approach. In the two-step approach, a composite is created before the actual analysis. In this case, a composite is typically created as a simple sum or average of observed variables. In the second step, the created composite scores are used as input in SEM. Although this approach is easy to implement, it has three clear drawbacks. Firstly, the creation of the composite is not part of the model, i.e., the relationships between a composite and its components are not modeled in the structural model, and consequently, this approach is susceptible to obfuscating model misspecifications, see also^13^. Secondly, this approach is limited to observed variables as components. Thirdly, this approach is limited to studying composites with preset weights, so-called fixed- weight composites. Consequently, the weights cannot be treated as free model parameters that are estimated. Another approach often found in the ecology literature is the one-step approach^23^. This approach specifies a composite as a dependent latent variable whose error term variance is fixed to zero. As a result, the composite and the relationships to its components are modeled, overcoming some of the limitations of the two-step approach^13^. However, researchers using the one-step approach face two other limitations. Firstly, since composites are always modeled as dependent latent variables, it is not possible to specify covariances between a composite and other variables. This is because covariances can only be specified between exogenous variables. An exogenous variable, as opposed to an endogenous variable, is a variable that is not explained in the model, i.e., there is no arrow pointing to it in the graphical model representation, see also [^24^, Chapter 3]. Thus, a dependent variable such as the composite in the one-step approach can never be an exogenous variable and therefore, no covariance between the composite and another variable can be specified. Secondly, since the variance of a composite is fully explained by the components, there is no variance left that could be explained by other variables in the model, i.e., it is not possible to specify the effects of other variables on the composite.

Given the limitations of the commonly used approaches to study composites in SEM, the goal of our study is to provide ecologists with an approach that allows them to model composites in SEM with the same flexibility they are accustomed to when modeling observed and latent variables. For this reason, we introduce the recently proposed Henseler–Ogasawara (H–O) specification to the field of ecology, which overcomes the limitations of the two-step approach and the one-step approach^25^. Specifically, it takes into account the formation of the composite and allows the modeling of composites as independent and dependent variables in the structural model.

The remainder of our study is structured as follows. In Section 2, we highlight the role of composites in the field of ecology by presenting various applications of composites. Furthermore, we show the approaches commonly used in the field of ecology to study composites in SEM, i.e., the two-step approach and the one-step approach, and discuss their merits and limitations. Subsequently, Section 3 introduces the H-O specification by elucidating its underlying mechanism and highlighting its advantages over established approaches. Section 4 illustrates the H–O specification and compares it with the approaches presented previously using an example from the field of ecology. Finally, we conclude our paper in Section 5.

## The role of composites in ecology and how they are currently being studied in SEM

Researchers using SEM can deal with three different types of variables: (i) observed variables, (ii) latent variables, and (iii) composites^23^. Observed and latent variables can be flexibly modeled in SEM. For example, they can be specified as independent and dependent variables, i.e., as a variable that affects or is affected by other variables, respectively. Moreover, when specified as exogenous variables, covariances with other exogenous variables can be specified. In contrast, flexible modeling of composites in SEM is still a challenge. This is because the commonly used one-step approach to model composites in SEM does not allow researchers to model effects on the composite or covariances between a composite and other variables, see Subsection 2.2 for an explanation. Thus, ecologists who study composites are currently at a disadvantage compared to those who study only observed and latent variables. This is particularly problematic for research in ecology, as composites are “often [..] both appropriate and necessary to represent the general ecological ideas that unify the study of communities and ecosystems” [^1^, p. 86]. In this section, we explain what composites are, including examples from ecology, and discuss common approaches to studying composites and their limitations.

### Composites in ecology

A composite is a weighted linear combination of variables, so-called components. Thus, forming composites is essentially a prescription for dimension reduction^26^. Composites are used to capture the collective effects of or on their components^14^. The components of a composite can be observed variables (e.g.,^27^), latent variables^13^, composites, or a mixture of the three (e.g., [^28^, Chapter 10]).

The literature distinguishes two types of composites, i.e., *fixed-weight composites* and *unknown-weight composites*^23^. The former refers to composites in which the relationships between the composite and its components, i.e., the weights, are fixed to certain values by a researcher according to some given a priori information. Usually, fixed-weight composites take the form of sums or averages, i.e., each component contributes equally to the composite. In contrast, the weights of an unknown-weight composite are not determined before the actual analysis; they are free model parameters and are estimated based on the composite’s relationships with other variables in the structural model. Therefore, the weights of unknown-weight composites are context specific.

In the ecology literature, composites loom large. For example, in plant ecology the plant community structure was modeled as a composite of four distinct plant biomass variables, i.e., plant biomass of C3 grasses, C4 grasses, herbaceous forbs and N-fixing legumes^27^. Similarly, in animal ecology, the grazing intensity was modeled as composite made up of recent and historic grazing intensity^29^. Table 1 provides further examples of composites from different fields of ecology.

**Table 1:** Examples of composites in ecology.

Ecology field	Composite	Components	SEM approach used	Reference
Plant ecology	Plant community structure	$\bullet$ Plant biomass of C3 grasses	Two-step approach	Antoninka et al.^27^
		$\bullet$ Plant biomass of C4 grasses		
		$\bullet$ Plant biomass of herbaceous forbs		
		$\bullet$ Plant biomass of N-fixing legumes		
	Light effect	$\bullet$ Light	One-step approach	Grace et al.^1^
		$\bullet$ Lightlog (natural logarithm of light)		
	Soil conditions	$\bullet$ Soil texture	Two-step approach	Verheyen et al.^30^
		$\bullet$ Soil moisture		
		$\bullet$ Soil pH		
	Land-use history and spatial isolation	$\bullet$ Secondary forest age	Two-step approach	Verheyen et al.^30^
		$\bullet$ Species-specific distances from colonization sources		
	Canopy structure	$\bullet$ Log-transformed basal area of the shrubs	Two-step approach	Verheyen et al.^30^
		$\bullet$ Cover of competitive herbs		
	Arbuscular mycorrhizal fungal abundance	$\bullet$ Mycorrhizal infection potential bioassay	Unclear	Chaudhary et al.^31^
		$\bullet$ Hyphal density		
		$\bullet$ Glomalin-related soil protein concentration		
		$\bullet$ Spore abundance		
	Abiotic conditions	$\bullet$ Soil salinity	PLS & SEM	Gough and Grace^32^
		$\bullet$ Flooding stress		
		$\bullet$ Percentage soil carbon		
	Abiotic factors	$\bullet$ Elevation	Two-step approach	Laughlin and Abella^33^
		$\bullet$ Soil pH		
		$\bullet$ Clay concentration		
		$\bullet$ Organic matter		
		$\bullet$ Soil nitrogen		
Animal ecology	Grazing intensity	$\bullet$ Recent grazing intensity by all herbivores	Two-step approach	Eldridge^29^
		$\bullet$ Historic grazing intensity by livestock		
	Predation risk	$\bullet$ Woody cover	One-step approach	Hopcraft^20^
		$\bullet$ Distance to drainages		
		$\bullet$ Landscape curvature		
	Macrohabitat type	$\bullet$ Lake (a type of wetlands)	One-step approach	Grace^34^
		$\bullet$ Impound (a type of wetlands)		
		$\bullet$ Swale (a type of wetlands)		
Global ecology	Climate	$\bullet$ Temperature	Two-step approach	Hu et al.^35^
		$\bullet$ Precipitation		
	Soil textural	$\bullet$ Carbon to nitrogen ratio	One-step approach	Jones et al.^19^
		$\bullet$ Percent loam		
		$\bullet$ Soil organic matter		
	Soil mineral	$\bullet$ Zinc (Zn) concentration	One-step approach	Jones et al.^19^
		$\bullet$ Iron (Fe) concentration		
		$\bullet$ Phosphorus (P) concentration		

### Common approaches to study composites in SEM

**Fig. 1:**
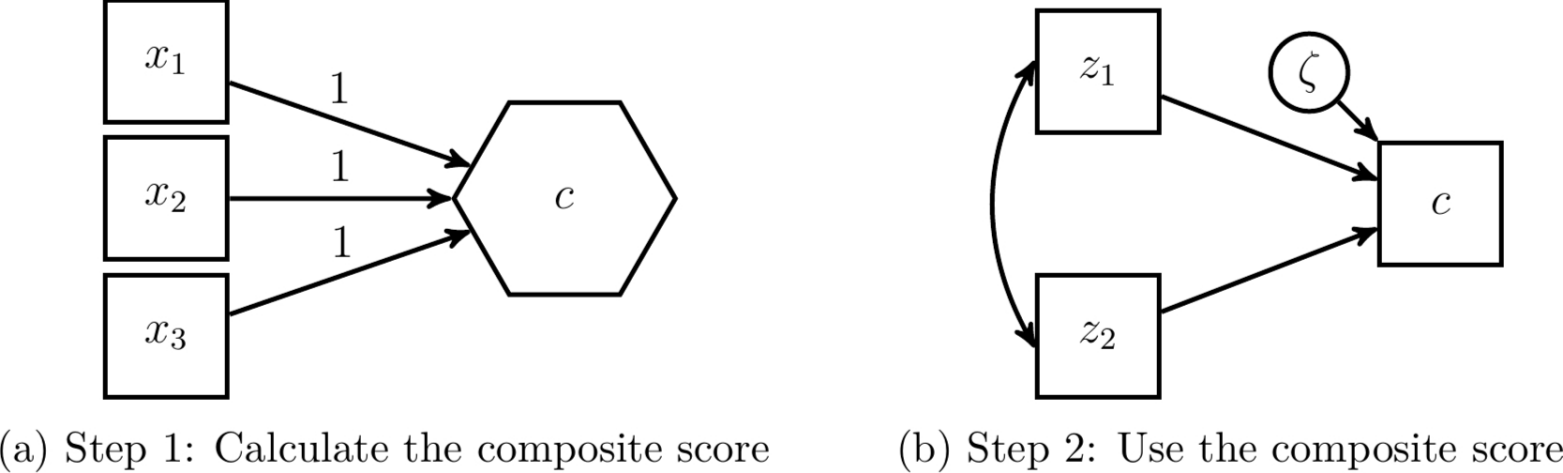
Studying a composite using the two-step approach.

Ecological researchers using SEM to study composites usually use one of the following two approaches (see also Table 1): (i) the two-step approach, or (ii) the one-step approach. The two-step approach is widely used in ecology (e.g.,^22,30^) and as its name suggests, it consists of two steps. As shown in Fig. 1a, in the first step, a composite score is calculated before the actual analysis. For this purpose, the components, i.e., the variables that make up the composite, are typically summed up or averaged. In our example from Fig. 1, the components $x_1,$ $x_2$ and $x_3$ are summed up to create the composite *c*. As is common in visualizations of structural equation models, the observed variables $x_1$, $x_2$ and $x_3$ are shown as rectangles, while composites are depicted as hexagons to distinguish them from latent variables, which are usually shown as ovals^23^. In the second step, the original components are replaced by the created composite score to perform the actual analysis. For instance, to examine the effects of two variables $z_1$ and $z_2$ on the composite *c*, the components $x_1$, $x_2$, and $x_3$ are replaced by *c*, i.e., the composite score, and only *c* is used in the analysis (see Fig. 1b).

Although the two-step approach is very appealing because of its ease of use, it suffers from several drawbacks. First, the composite is not modeled as it is created before the actual analysis. As a result, researchers cannot leverage the full capacities of SEM. For instance, the available information to estimate, assess and compare the model is reduced because the original components are replaced by the composite score. Similarly, model misspecification can be masked because the formation of the composite remains unassessed with regard to overall model fit assessment. In addition, researchers cannot take advantage of SEM’s direct maximum likelihood approach to handle missing values^36^. Second, the weights are not considered as model parameters and therefore cannot be estimated. Consequently, the two-step approach can only be used to study fixed-weight composites. Third, the two-step approach is limited to composites formed only from observed variables. Therefore, it is not possible to study composites involving latent variables such as done in Frauendorf et al.^13^, who studied a body condition index which was modeled as a composite involving the latent variable size-corrected mass among others.

**Fig. 2:**
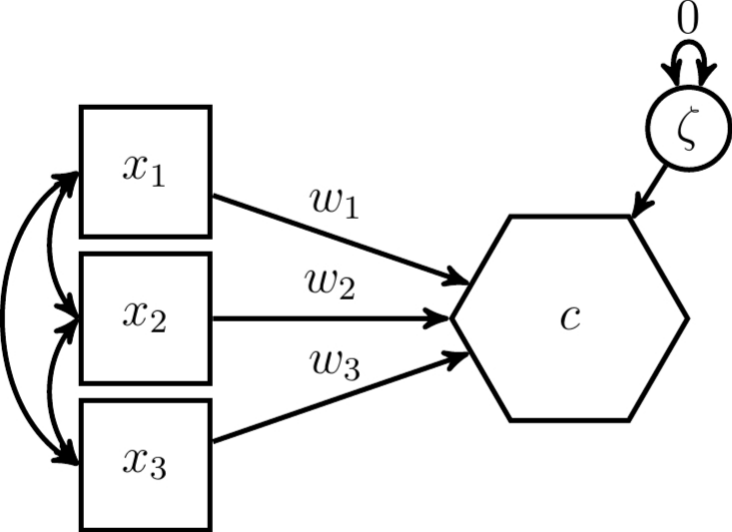
Modeling a composite using the one-step approach.

A more sophisticated alternative to the two-step approach is the one-step approach. It was introduced to ecologists by Grace and Bollen^23^ and, unlike the two-step approach, the one-step approach models the composite in the structural equation model. As shown in Fig. 2, the one-step approach models a composite as a dependent latent variable, with the variance of the error term fixed to 0. Therefore, the composite *c* in Fig. 2 could just as well be drawn as an oval. Note that in the case of a composite with unknown weights, the composite must affect at least one other variable, and the variance of the composite must be determined, e.g., by fixing one of the weights, to ensure that the model parameters are identified, i.e., to ensure that there is a unique solution for the model parameters^11^. For more information on model identification in the case of the one-step approach, the interested reader is referred to MacCallum and Browne^37^.

The one-step approach overcomes some drawbacks of the two-step approach because it explicitly models the composite in the structural model. As a consequence, the complete information is used for the model estimation and assessment, SEM’s direct maximum likelihood approach can be used to deal with missing values^36^, and composites composed of latent variables can be modeled. However, this approach has new drawbacks. Particularly, because a composite is always modeled as a dependent latent variable whose error term’s variance is fixed to 0, it is not possible to model effects of other variables on the composite, next to its components^37^. In this case, the components together with the other independent variables would make up the composite – a situation not intended by the researcher. Hence, it is not possible to use the one-step approach to model a composite that captures the collective effect on its components. For example, Verheyen et al.^30^ investigated the effect of soil condition on the canopy structure. Both are considered as composites. With the one-step approach, it is not possible to model this relationship. This may explain why the authors used the two-step approach in their original study. Similarly, the one-step approach does not allow us to specify covariances between the composite and other exogenous variables of the model. Since the composite is always treated as dependent variable, at its best, covariances between the composite’s error term and the other exogenous variables could be specified. However, since the variance of the error term must be fixed to 0 to ensure that the composite is fully made up of its components, this is a fruitless endeavor. For example, Verheyen et al.^30^, Fig. 1 proposed a covariance between soil condition and past land use in their conceptual model. Both soil condition and past land use are considered as composites. Using the one-step approach, it is not possible to specify a covariance between the two composites.

To conclude, composites are frequently encountered in the ecological literature. However, the commonly used two-step and one-step approaches have limitations. While the two-step approach does not model the components of the composite and is thus prone to model misspecification, the one-step approach is limited in its modeling flexibility. As a result, ecologists using the one-step approach may have difficulty adequately representing their conceptual models with a statistical model.

## The Henseler–Ogasawara specification

In this section, we present the recently proposed Henseler–Ogasawara (H–O) specification, which allows researchers to model composites with the same flexibility as they are accustomed to when modeling latent and observed variables^25^. The H–O specification was sketched by Henseler^28^ and draws from Ogasawara^38^’s idea to conduct a canonical correlation analysis in SEM, hence the name of the specification. Subsequently, the H–O specification was refined to reduce its complexity^39^.

**Fig. 3:**
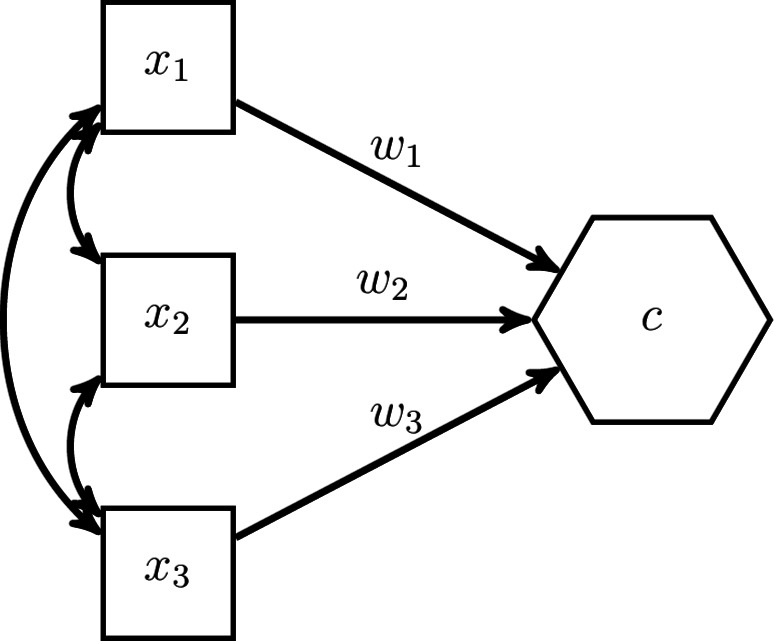
Illustration of a composite expressed in terms of weights.

The starting point is a composite *c* composed of a set of components ${\varvec{x}}$:

1 $$\begin{aligned} c = {\varvec{w}}' {\varvec{x}} , \end{aligned}$$

where ${\varvec{w}}$ contains the weights to form the composite. Although the H–O specification allows for observed variables, latent variables and/or composites to be components of a composite, we limit ourselves to observed variables in this section. Fig. 3 illustrates the situation in which three observed variables $x_1$, $x_2$, and $x_3$ make up a composite *c*. Forming a composite does not impose any restrictions on the covariances of the components, as indicated by the double-headed arrows.

**Fig. 4:**
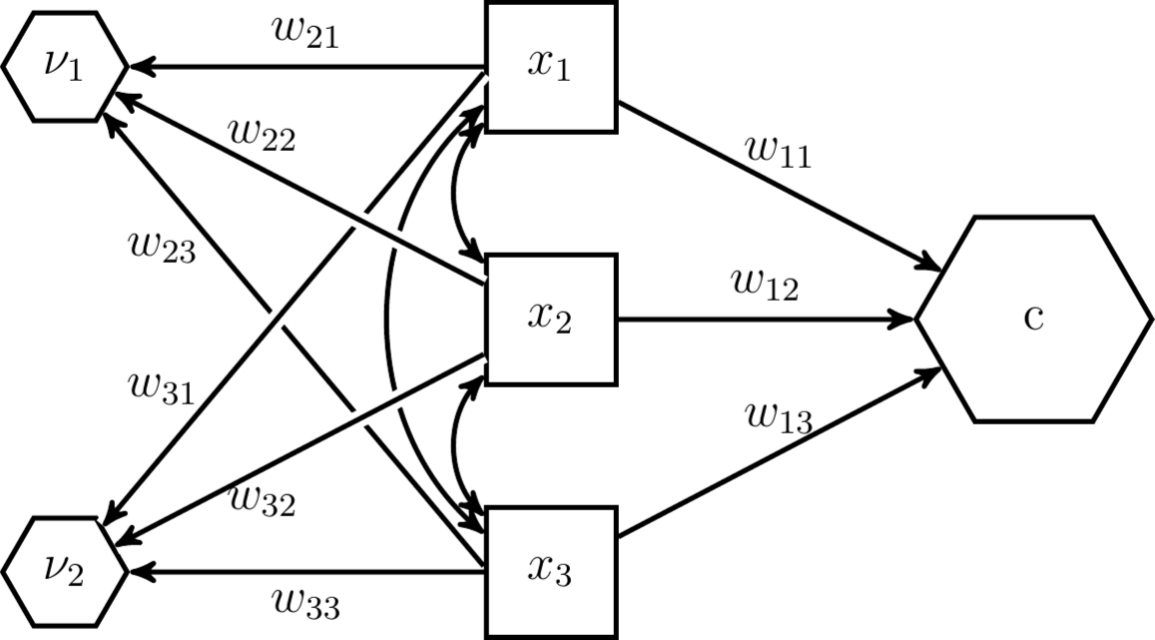
The H–O specification creates as many composites as components.

In the H–O specification not just one composite, but as many composites as components are extracted from the set of components ${\varvec{x}}$:

2 $$\begin{aligned} \begin{pmatrix} c \\ {\varvec{\nu }} \end{pmatrix} = {\varvec{W}}' {\varvec{x}} \end{aligned}$$

where *c* is the composite of interest, ${\varvec{\nu }}$ are the additionally extracted composites that capture the remaining covariances between the components that are not explained by the composite of interest *c*, and the quadratic matrix ${\varvec{W}}$ contains in its columns the weights used to form these composites. We follow Henseler^28^ and name the additionally extracted composites excrescent variables. Fig. 4 illustrates this idea for our example with three components. As can be seen, next to the composites of interest *c*, two excrescent variables $\nu _1$ and $\nu _2$ are constructed from the three components $x_1$, $x_2$ and $x_3$.

**Fig. 5:**
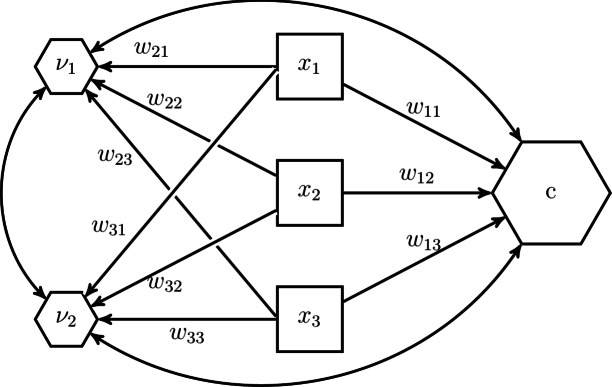
The H–O specification expresses the relationship between the components and composites in terms of composite loadings.

In addition to extracting not only the composite, the H–O specification expresses the relationships between the composites and their components in terms of composite loadings instead of weights as shown in Fig. 5.

This can be achieved by transforming Eq. 2 as follows:

3 $$\begin{aligned} {\varvec{x}} = ({\varvec{W}}')^{-1} \begin{pmatrix}c \\ {\varvec{\nu }} \end{pmatrix} = {\varvec{\Lambda }} \begin{pmatrix}c \\ {\varvec{\nu }} \end{pmatrix}, \end{aligned}$$

where the quadratic matrix ${\varvec{\Lambda }}$ contains the composite loadings. The advantage of expressing the relationship between composites and their components in terms of composite loadings is that the composite *c* can be embedded in a larger structural model as an exogenous or an endogenous variable. Note that expressing the relationships between the composites and their components in terms of composite loadings is only possible if the weight matrix ${\varvec{W}}$ is not singular, i.e., the composites are not perfectly linearly dependent (It is also assumed that the components making up the composites are not perfectly linearly dependent). We will discuss how to achieve this in the next paragraph.

**Fig. 6:**
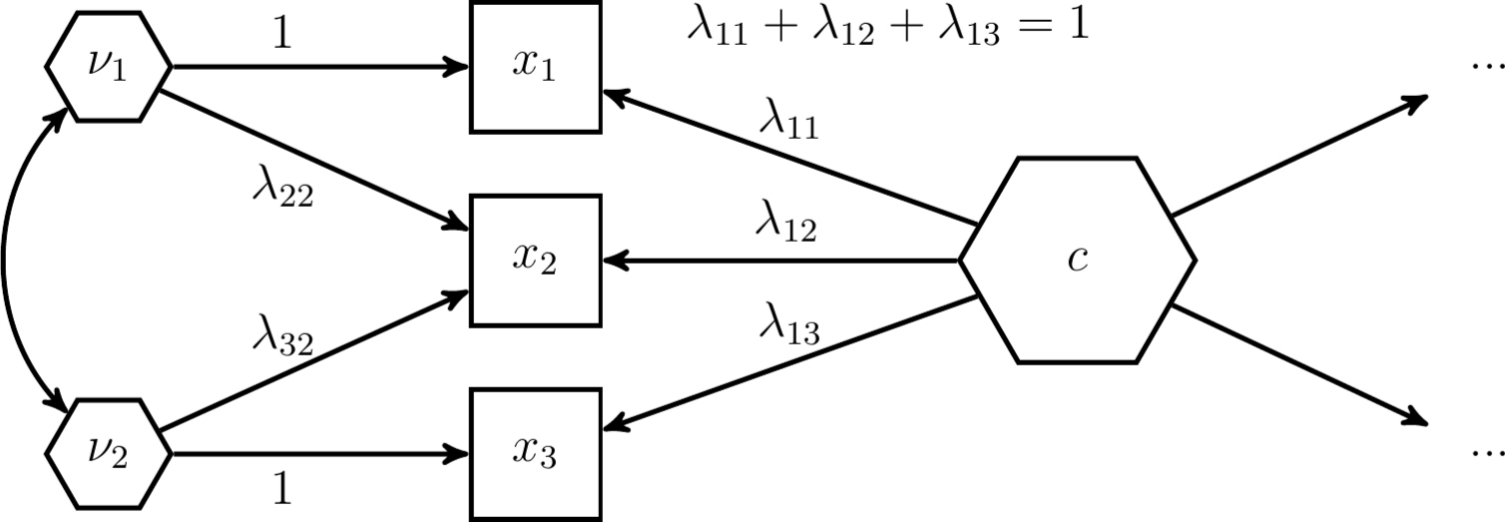
H–O specification with free weights.

The parameters of the model shown in Fig. 5 are not identified, i.e., there exists no unique solution for them. To address this issue, parameters need to be constrained. While there are several ways of how to constrain the parameter such that the model becomes identified, we follow the constraints proposed in the refined H–O specification to ensure model identification^39^, see also Table 2. Fig. 6 illustrates the constraints for our three-components example. In the *Quantitude* podcast, Gregory Hancock and Patrick Bauer coined the term “handshake specification” for this kind of specification (https://quantitudepod.org/s5e08-confirmatory-composite-analysis/).

First, the variances of the composites, i.e., the composite of interest *c* and the excrescent variables ${\varvec{\nu }}$ must be determined. The literature on latent variable models offers various methods that can be used for that purpose, e.g., the reference variable method, the variance standardization method or the effects coding method (see, e.g., [^24^, Chapter 14]). In the model displayed in Fig. 6, the effects coding method was used for the composite of interest, while the reference variable method was applied for the excrescent variables. As a result, the sum of loadings of the composite of interest is set to 1 and the composite loadings of $x_2$ on $\nu _1$ and $x_3$ on $\nu _2$ are fixed to 1. It is important to note that each observed variable is only allowed to serve as a reference variable once. Next to fixing the scales of the composites, additional loadings need to be constrained. Following the refined H–O specification, only two observed variables are allowed to load on one excrescent variable^39^. Hereby, it has to be ensured that no excrescent variables are connected to exactly the same observed variables and that no observed variable loads on more than two excrescent variables. Moreover, the excrescent variables have to be uncorrelated with the composite of interest. This ensures that the composites are not linearly dependent. Further, if the weights are to be freely estimated, i.e., an unknown-weight composite is studied, the composite of interest must be related to at least one other variable in the model in addition to its components as highlighted by the omission points in Fig. 6. Note, by default, most SEM implementations specify the components as random measurement error contaminated variables. If this is the case, the variances of these error terms need to be constrained to 0 to ensure that the composites are fully composed of their components.

**Table 2:** Identification rules used in the refined H–O specification.

	Free weights (Unknown weights composite)	Unit weights (Fixed weights composite)
(1) Rule	Fix the sum of the loadings of the composite of interest to 1 (effects coding method), fix one of the loadings of the composite of interest to 1 (reference variable method) or fix the variance of the composite of interest to 1 (variance standardization method).	Fix the sum of the loadings of the composite of interest to 1 (effects coding method).
(2) Rule	Fix all loadings per excrescent variable to 0 except two of them. No excrescent variable is allowed to be connected to the exact same two components and no component is allowed to load on more than two excrescent variable.	
(3) Rule	Fix one of the two remaining loadings per excrescent variable to 1 (reference variable method). No component is allowed to serve several times as reference variable.	Fix the remaining loadings per excrescent variable in such a way that their sum equals 0, e.g., to -1 and 1.
(4) Rule	Fix the covariances between the excrescent variables and the composite of interest to 0.	
(5) Rule	Connect the composite of interest with at least one other variable of the model besides its components.	

This parameterization of the H–O specification shows several characteristics. First, the composite of interest, together with the excrescent variables, completely spans the space of the components. In particular, the excrescent variables account for the covariances between the components that cannot be explained by the composite of interest. As a consequence, the H–O specification does not impose any constraints on the variances of the components and their covariances, i.e., the model perfectly replicates the variance-covariance matrix of the components. Second, the weights are freely estimated. Third, the composite of interest accounts for all the covariances between the components and other variables in the model. In the literature such a composite is also known as *emergent variable*^40,41^. In other words, all information between the components and other variables in the model is conveyed by the composite of interest^42^. In the following, we will show how some of the assumptions can be relaxed.

**Fig. 7:**
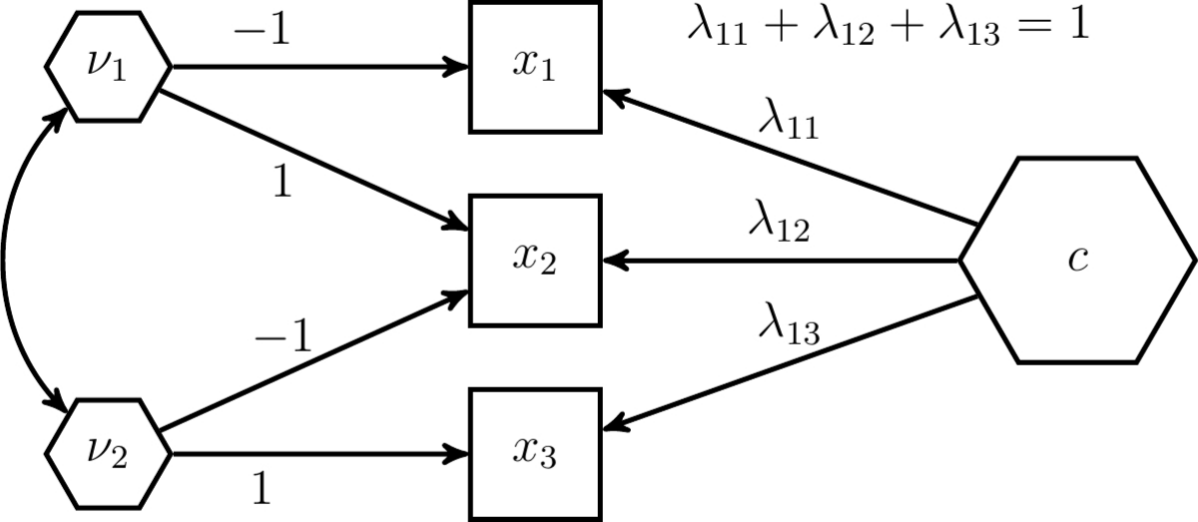
The H–O specification with unit weights.

Besides unknown-weight composites, there are also situations in which researchers study fixed-weight composites, i.e., the weights used to form the composite of interest are determined by the researcher prior the analysis. Arguably, the most common fixed-weight composites appear as sums or averages of their components. To model such composites in the H–O specification, further parameter constraints need to be imposed^21^. First, the composite loadings of each excrescent variable need to be fixed in such a way that their sum equals 0. Second, by considering the effects coding method to fix the scale of the composite of interest, we can determine whether the resulting composite of interest will be the sum or the average of its components. In particular, if the sum of the composite loadings is fixed to 1, the resulting composite of interest will be the sum of its components, and if the sum of the loadings is fixed to the number of components, the composite of interest will be the average of its components. Fig. 7 shows our example model from Fig. 6 where unit weights are used instead of free weights to form the composite of interest *c*. Note, in contrast to unknown-weight composites, the composite of interest does not need to be related to any other variable in the model to ensure that the related model parameters are identified. Moreover, Table 2 summarizes the constraints necessary to achieve unit weights in the H–O specification.

In the two H–O specifications presented previously, i.e., the H–O specification with free weights and the H–O specification with equal weights, we assumed that the composite of interest accounts for all the covariances between the components and other variables in the model. In some situations, this assumption may be too restrictive and researchers would like to relax it. For instance, if the components are not expected to show conceptual unity^17^. An example is provided by Grace and Bollen^23^ where the components of two different composites of interest are allowed to be freely correlated, i.e., their covariances are not necessarily fully accounted for by the two composites of interest. To achieve such a situation in the H–O specification, we can specify covariances between the excrescent variables and other variables of the model, see also Section 4.

To estimate the model parameters, researchers can, in principle, use any SEM implementation, such as the R package lavaan^43^, or the commercial software Mplus^44^ and Amos^45^, and their implemented estimators, such as the full-information maximum-likelihood estimator^46^ and the generalized least squares estimator^47^. However, the weights are not computed directly because the H–O specification expresses the relationships between the composites and their components in terms of composite loadings instead of weights. To address this issues, researchers can exploit the feature of most SEM implementations to specify new parameters. Specifically, researchers can specify the weights as elements of the inverted composite loading matrix as shown in Eq. (3). Similarly, the standardized weights can be obtained by multiplying the unstandardized weight with the ratio of the standard deviation of the component and the standard deviation of the composite. For more details, see for example Schamberger et al.^48^. Since SEM implementations have been developed for latent variable models, the starting values for the composite loadings are often chosen suboptimally, which can lead to a non-convergence of the estimation. It has been shown that starting values of 0 for the free composite loadings are often a good choice.

## Illustrative example

### Description

In this section, we showcase the H–O specification. In particular, we use the empirical study of Verheyen et al.^30^ to illustrate its flexibility and advantages over commonly used approaches to study composites. In one part of their study, SEM was used to investigate the relationships between past land use (*Land*), soil condition (*Soil*), canopy structure (*Cano*), and colonization frequency (*Colo*). In their original study, *Land*, *Soil* and *Cano* were modeled as composites, while *Colo* was modeled as a single-indicator latent variable. To estimate the relationships between the four variables, the two-step approach was used, i.e., in the first step the composites were created as sums of their components, and subsequently the resulting composite scores were used to estimate the parameters of the structural model. For more details on the scope of their study and the original analysis, we refer to Verheyen et al.^30^.

**Fig. 8:**
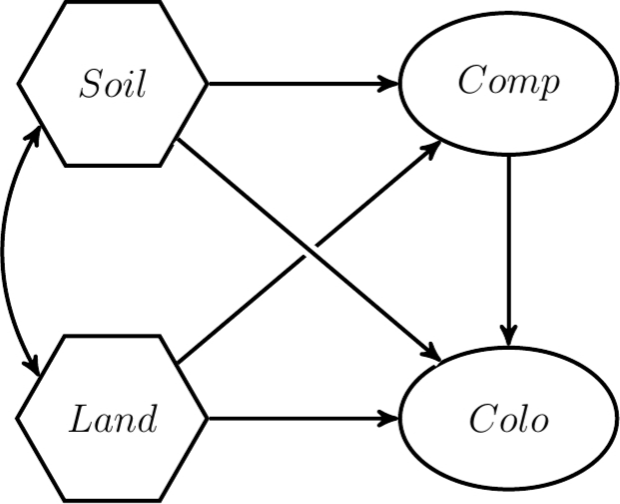
Conceptual model adopted from Verheyen et al.^30^.

The original dataset used in Verheyen et al.^30^ is not publicly available. Therefore, in our illustrative example, we use the selective subset of the dataset as studied in Grace and Bollen^23^ to introduce the one-step approach. This dataset consists of 180 forest stands, ranging in age from 1 to 195 years, and seven variables. The latter comprises the estimated age of a forest stand (*age*), the distance from a forest stand to the nearest source patch (*dist*), the soil texture (*text*), the soil moisture (*mois*), the soil pH (*pH*), the cover of competitive herbs (*cover*), and the frequency of the colonizing forest herbs (*colf*). The herb species whose colonization is considered in this illustrative example is Lamium galeobdolon. The correlation matrix of the variables, including their standard deviations, is publicly available; see Table 1 in Grace and Bollen^23^. For more details on the dataset, we refer to the studies of Verheyen et al.^30^ and Grace and Bollen^23^. The variables *text*, *mois* and *pH* are used as components to make up *Soil*, while *Land* is composed of the components *age* and *dist*. In addition, *colf* is used as an indicator to measure *Colo*. Furthermore, and in contrast to the original study by Verheyen et al.^30^, we follow Grace and Bollen^23^ and replace *Cano* with competitor abundance (*Comp*) modeled as a latent variable using *cover* as single indicator. Arguably, Grace and Bollen^23^ made this adjustment because, in contrast to the H–O specification, the one-step approach they introduced cannot be used to model effects on a composite. Moreover, we assume that 10% of the variances in *colf* and *cover* are due to random measurement error, as Grace and Bollen^23^ did. Therefore, we fix the variances of the two random measurement errors to 0.009102 and 0.003486, respectively. To take into account a given reliability of an indicator, we can fix the variance of its corresponding random measurement error to: $(1-\text {rel}(x)) \times \text {var}(x)$ [^49^, Equation 7-6], where $\text {rel}(x)$ is the assumed reliability of the indicator and $\text {var}(x)$ is the variance of the indicator. For our two single indicators with an assumed reliability of 0.9, the variances of the random measurement errors need to be fixed to 0.009102 $(= (1-0.9) \times 0.3017^2)$ and 0.003486 $(= (1-0.9) \times 0.1867^2)$, respectively. The standard deviations of 0.3017 and 0.1867 were reported in the study of Grace and Bollen [^23^, Table 1]. The conceptual model is sketched in Fig. 8. It resembles the conceptual model studied by Verheyen et al. [^30^, Fig. 1] with the difference that *Cano* is replaced by *Comp*.

### Model specification

**Fig. 9:**
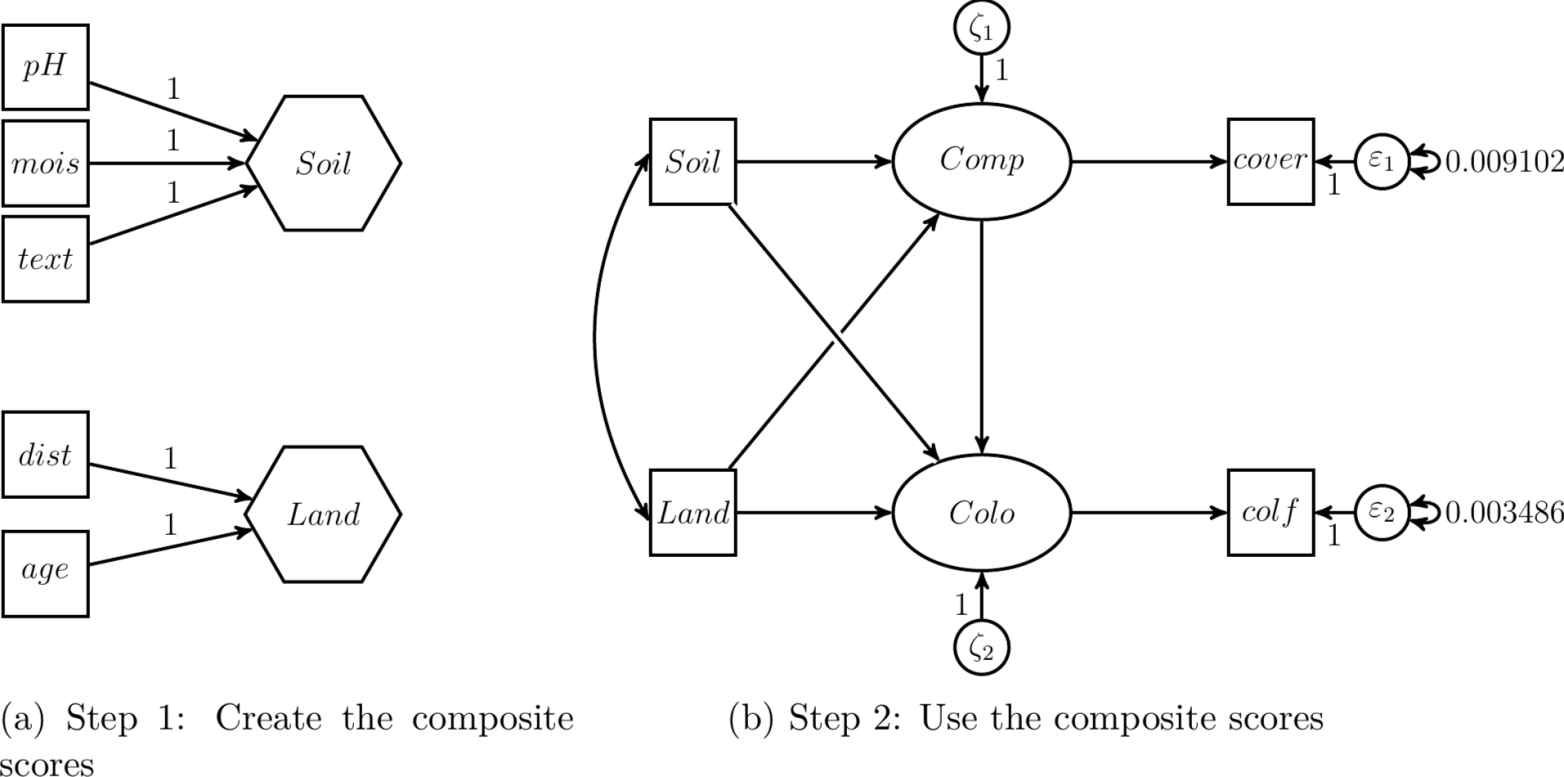
The two-step approach used in the illustrative example.

As a starting point, we use the two-step approach as originally employed by Verheyen et al.^30^ to estimate the model from Fig. 8. In the first step, the scores for the composites *Soil* and *Land* are calculated by summing up their respective components, i.e., *text*, *mois* and *pH*, and *age* and *dist*. Subsequently, in the second step, these scores are used as input to estimate the structural model. *Comp* and *Colo* are modeled as single-indicator latent variables. The two-step approach is illustrated in Fig. 9.

**Fig. 10:**
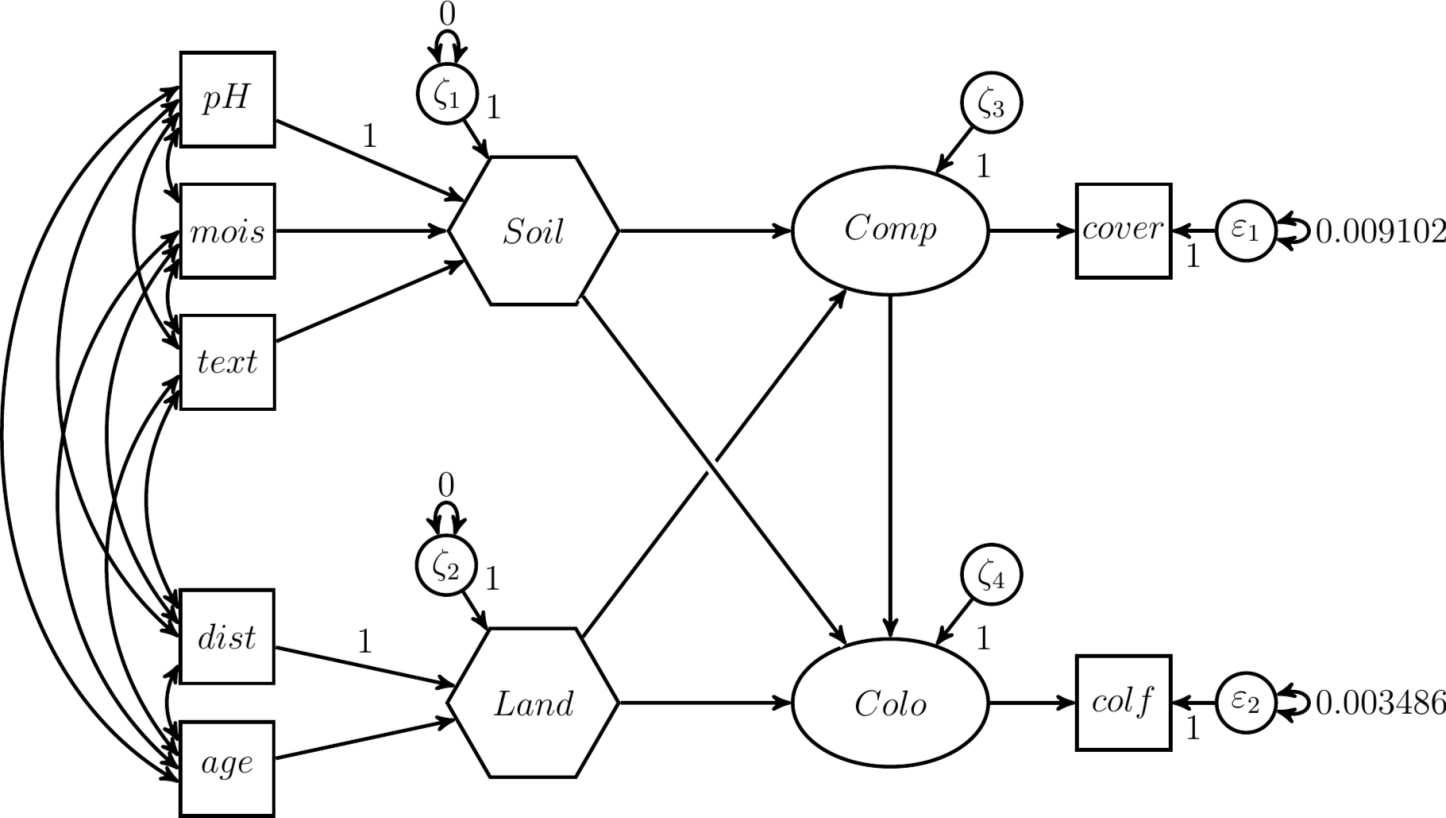
One-step approach as proposed by Grace and Bollen^23^.

Moreover, we use the one-step approach as employed in Grace and Bollen^23^. The model specification used is shown in Fig. 10.

As can be seen, *Soil* and *Land* are modeled as dependent latent variables with the variances of their error terms fixed to 0. Although this renders the latent variables into composites, this specification does not allow directly specifying a covariance between *Soil* and *Land* as originally proposed in the conceptual model (see Fig. 8). Instead, the covariance between *Soil* and *Land* is replaced by covariances between the components of *Soil* and *Land*. As a consequence, the covariances between the two sets of components are not fully accounted for by *Soil* and *Land*. Moreover, the weights of *pH* and *dist* are fixed to 1 to determine the variance of the two composites, *Soil* and *Land*.

**Fig. 11:**
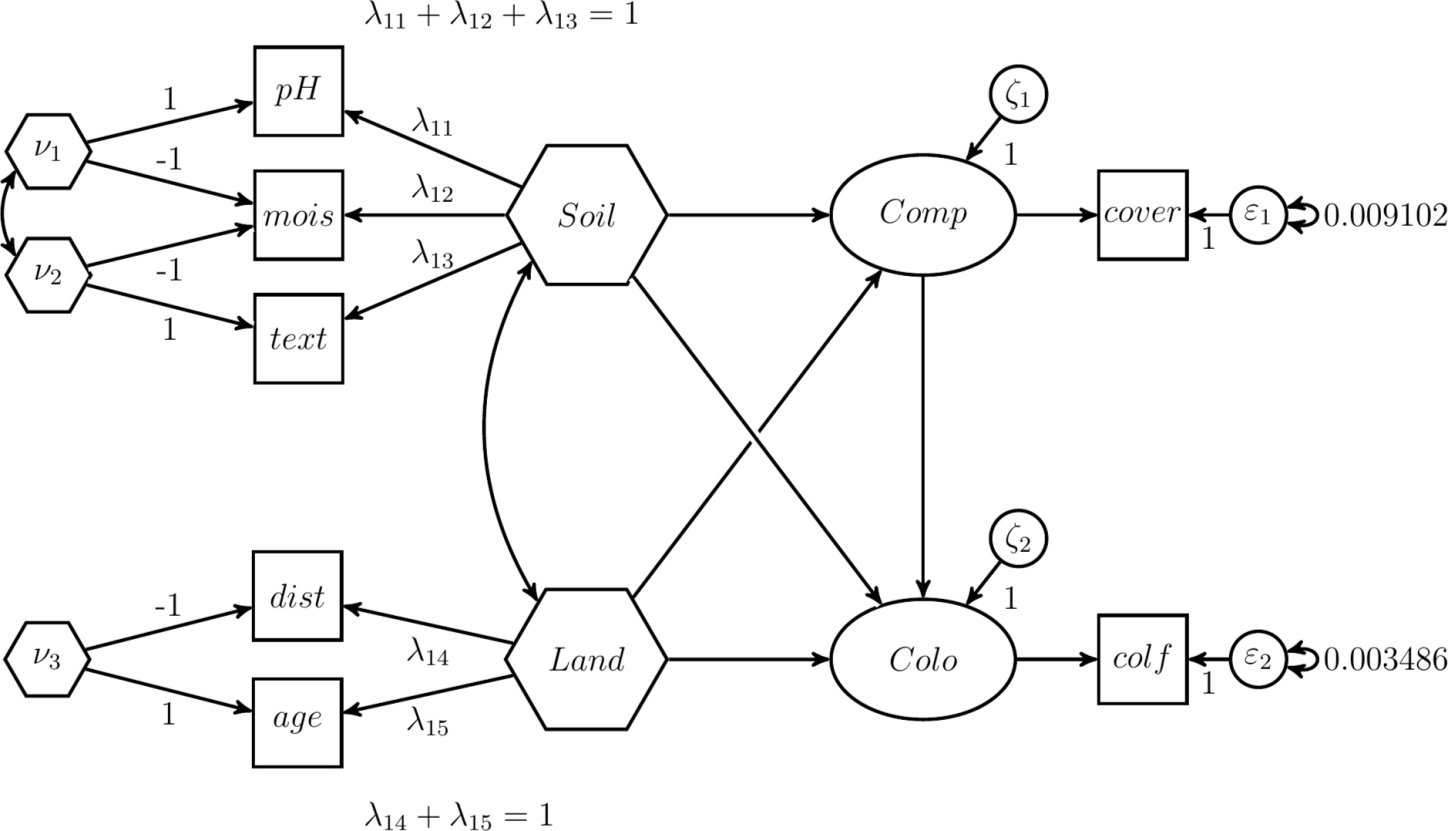
H–O specification mimicking the two-step approach.

Finally, we present three H–O specifications. The first H–O specification mimics the results of the two-step approach, i.e., it produces the same parameter estimates. For this purpose, composites with unit weights need to be specified in the H–O specification, i.e., fixed weights composites are modeled. Fig. 11 depicts the H–O specification to model *Soil* and *Land* as sums of their components.

As can be seen in Fig. 11, not just one composite, but as many composites as components are extracted from a set of components. Specifically, in addition to the composite of interest *Soil*, two excrescent variables $\nu _1$ and $\nu _2$ are constructed from the three components *text*, *mois* and *pH*. Similar is done for the components *age* and *dist* forming the composite *Land*. Moreover, as discussed in the previous section, we employ the effects coding method to determine the variances of the composites *Soil* and *Land*. In particular, we fix the sum of the composite loadings to 1. In addition and to ensure that *Soil* and *Land* are the sum of their components, we set the composite loadings of each excrescent variable in such a way that their sum is equal to 0. In our case, we fix the composite loadings of each excrescent variable to 1 and -1.

**Fig. 12:**
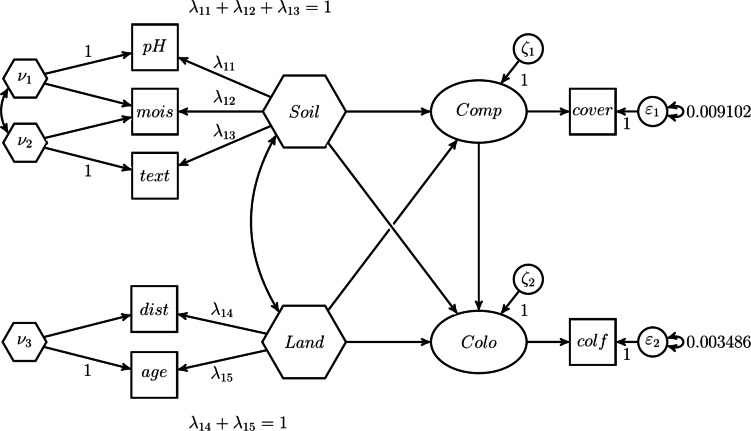
H–O specification to model unknown weights composites.

The H–O specification can also be used to model unknown-weight composites, i.e., the weights of the composites of interest are freely estimated. This highlights one of the advantages of the H–O specification over the two-step approach where it is not possible to freely estimate the weights. For that purpose, compared to the first H–O specification, we free one of the composite loadings per excrescent variable in our second H-O specification. As shown in Fig. 12, the constraints on the composite loadings of *mois* on $\nu _{1}$, *mois* on $\nu _{2}$, and *dist* on $\nu _{3}$ are removed, allowing these loadings to be freely estimated. This is the model that comes closest to the conceptual model, see Fig. 8.

**Fig. 13:**
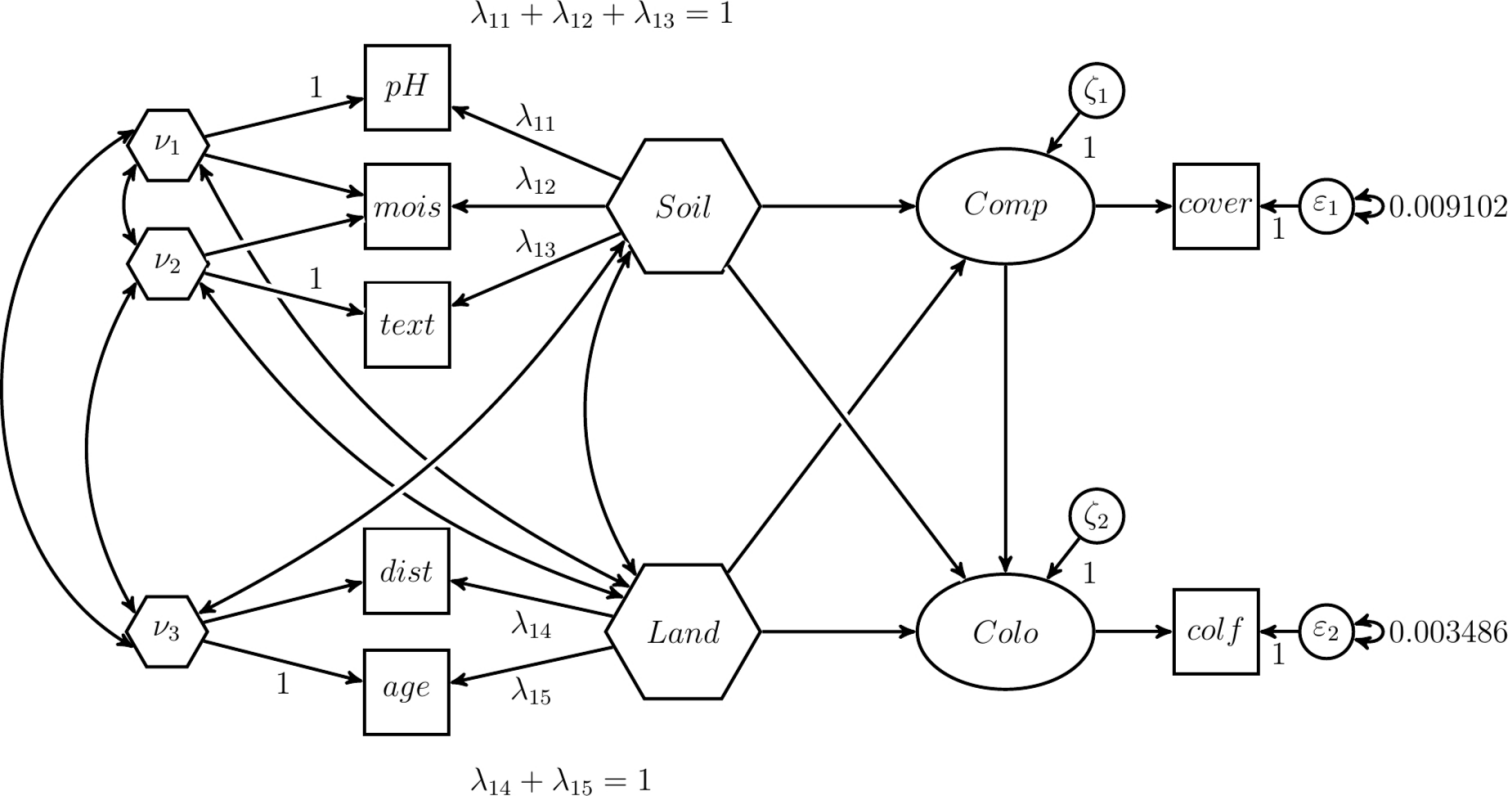
H–O specification with free weights which mimics the one-step approach used in the illustrative example.

To mimic the one-step approach used in the illustrative example with the H–O specification, we specify a model similar to the one shown in Fig. 12. To relax the assumption that all covariances between the two sets of components are accounted for by *Soil* and *Land*, we need to specify covariances among the excrescent variables and between the excrescent variables and *Land* and *Soil*, as illustrated in Fig. 13. The excrescent variables not only capture the variation in the corresponding components that is not extracted by the composite of interest, but also provide the flexibility to capture the remaining covariance between the components when analysts do not want to impose a particular structure on them.

### Results

To estimate the parameters of the models specified above, we use the maximum-likelihood estimator as implemented in the R package lavaan^43^. All estimations terminated normally. The unstandardized weights and standardized path coefficient estimates, including their standard errors and the model fit statistics, are reported in Table 3. We deliberately report the standardized path coefficient estimates to provide a more meaningful comparison of the different approaches, as the unstandardized path coefficient estimates can differ in magnitude when the weights are freely estimated due to a different scaling of the composites of interest. For the two-step approach, the model fit statistics are omitted as they are based on a different variance-covariance matrix and are therefore not informative for our comparison. Note,  a H–O specification was presented that not only reproduces the parameter estimates of the two-step approach, but also its fit statistics^21^. To obtain the weight estimates in the case of the H–O specifications, we make use of the lavaan feature to specify new parameters. Specifically, the unstandardized weight estimates are obtained as the elements of the inverted loading matrix. The complete R code is accessible via the following URL: https://osf.io/c7jz2/?view_only=e4722965ce3b4ffbb0890a2d55868d93.

**Table 3:** Results for our illustrative example.

Weights	Two-step		H–O (unit weights)		H–O (free weights)		One-step		H–O (relaxed)	
	Est	SE	Est	SE	Est	SE	Est	SE	Est	SE
$w_\text {pH}$	1.000	N.A.	1.000	N.A.	0.565	0.301	1.000	N.A.	1.323	0.298
$w_\text {mois}$	1.000	N.A.	1.000	N.A.	1.088	0.180	0.234	0.282	0.309	0.384
$w_\text {text}$	1.000	N.A.	1.000	N.A.	1.031	0.574	1.323	0.829	1.750	0.722
$w_\text {dist}$	1.000	N.A.	1.000	N.A.	0.193	0.062	1.000	N.A.	0.175	0.066
$w_\text {age}$	1.000	N.A.	1.000	N.A.	-0.740	0.085	-4.222	1.445	-0.739	0.090
Standardized path coefficients										
Soil $\rightarrow$ Comp	0.428	0.067	0.428	0.067	0.284	0.076	0.329	0.069	0.329	0.069
Soil $\rightarrow$ Colo	-0.101	0.077	-0.101	0.077	-0.016	0.065	0.018	0.064	0.018	0.064
Land $\rightarrow$ Comp	-0.049	0.074	-0.049	0.074	0.266	0.076	0.295	0.074	0.295	0.074
Land $\rightarrow$ Colo	-0.361	0.066	-0.361	0.066	-0.674	0.053	-0.682	0.050	-0.682	0.050
Comp $\rightarrow$ Colo	-0.320	0.077	-0.320	0.077	-0.114	0.068	-0.122	0.070	-0.122	0.070
Soil $\leftrightarrow$ Land	0.232	0.071	0.232	0.071	0.425	0.061	N.A.	N.A.	0.289	0.101
Fit statistics										
$\chi ^2$ statistic	Not		137.683		39.577		6.888		6.888	
p-value (*df*)	informative		$<0.01$ (11)		$<0.01$ (8)		0.076 (3)		0.076 (3)	
RMSEA			0.253		0.148		0.085		0.085	
SRMR			0.175		0.058		0.021		0.021	
CFI			0.654		0.914		0.989		0.989	
TLI			0.340		0.774		0.926		0.926	
AIC			1518.938		1426.831		1404.143		1404.143	
BIC			1573.218		1490.691		1483.967		1483.967	
SABIC			1519.379		1427.351		1404.792		1404.792	

Table 3 shows that the H–O specification using unit weights does indeed produce unstandardized weights of 1 for the composites *Soil* and *Land*. Therefore, it also produces the same path coefficient estimates as the two-step approach. In contrast to the two-step approach, the H–O specification allows us to assess the fit of the model including the components of the composites. This information is not available for the two-step approach because it replaces the components with the corresponding composite scores. As highlighted in Table 3, the fit measures commonly considered to judge the overall model fit such as the $\chi ^2$ test, the root mean square error of approximation [RMSEA, ^50^], and the standardized root mean square residual [SRMR, ^51^], indicate that the model does a poor job of describing the covariances between the observed variables. Since the structural model is saturated, this may be due to a suboptimal weighting of the components or to the fact that the two composites, *Soil* and *Land*, cannot fully account for the covariances between the two sets of components and their effects on the outcome variables. Therefore, in a next step, we consider the results of the H–O specification with free weights.

In the H–O specification with free weights, the weights are chosen in such a way that the covariances between the components and the other observed variables are best replicated. Although the fit measures and information criteria such as Akaike information criterion [AIC, ^52^] Bayesian information criterion [BIC, ^53^], sample-size-adjusted BIC [SABIC, ^54^], improved compared to H–O specification with unit weights and the H–O specification with unit weights shows a significantly worse fit ($\Delta \chi ^2 = 98.107, df=3, p<0.01$), the fit measures still indicate a substantial misfit of the H–O specification with free weights. Consequently, this model is likely not a good description of the data at hand.

In the last H–O specification, we relax the assumption of the previous two H–O specifications that all the covariances between the two sets of components are accounted for by the two composites *Soil* and *Land*, as done in the one-step approach proposed by Grace and Bollen^23^. As shown in Table 3 this H–O specification produces the identical results as the one-step approach, i.e., standardized path coefficient estimates and fit statistics. Note, the unstandardized weights are different because the variances of the composites *Soil* and *Land* are determined differently. Moreover, the one-step approach does not allow us to adequately represent the conceptual model (Fig. 8) as it is not possible to specify the covariance between the two composites *Soil* and *Land*. Compared to the H–O specification with free weights, the fit measures and information criteria are improved. The $\chi ^2$ test for exact model fit indicates no significant misfit, and the $\chi ^2$ difference test indicates that the H–O specification with free weights fits the data significantly worse than this H–O specification ($\Delta \chi ^2=32.689, df=5, p<0.01$).

## Discussion

This study introduces the H–O specification to the field of ecology. The H–O specification allows researchers to model composites in SEM with the same flexibility they are accustomed to when modeling observed and latent variables, and it overcomes the drawbacks of the approaches commonly used in ecology to study composites. In particular, the H–O specification allows modeling of composites with fixed and unknown weights. In addition, it is possible to specify the effects of other variables on the composite. Thus, the H–O specification overcomes the limitation of the one-step approach, where it is not possible to specify effects of other variables on the composites. In this way, it is possible to model not only a collection of effects on an outcome variable, but also a collection of effects on the components. Moreover, the H-O specification models the composite including its components and thus allows for more in-depth model assessment such as model comparison using the $\chi ^2$ difference test, compared to the two-step approach. We highlight the flexibility of the H–O specification using an illustrative example that was also used to introduce the one-step approach to ecological researchers. Our results show that the H–O specification can mimic the results of the other approaches.

In our presentation of the H–O specification, we assume that all the components are observed variables and thus free from random measurement error. If this assumption does not hold, the parameter estimates associated with the composites will be biased due to attenuation, which can lead to questionable conclusions drawn from the model, e.g.,^55,56^. To address this issue and to account for random measurement error in the components, the components can be modeled as latent variables with observed variables as their measures^21^. In this case, a composite would be made up of latent variables, e.g.,^57^.

We limit the scope of our study to the two- and one-step approach to study composites. However, there are other approaches that have been proposed to study observed variables, latent variables and composites such as consistent partial least squares [PLSc, ^58^] and integrated structured component analysis [IGSCA, ^59^]. For example, Gough and Grace^32^ used partial least squares^60^ to obtain composite scores. However, to use these approaches, researchers need to leave the SEM framework and use other statistical software. Moreover, next to the one-step approach, there are approaches in SEM that allow for modeling composites such as the pseudo-indicator approach^61^. However, all of these approaches are rarely, if ever, used in the field of ecology. Therefore, these approaches have been excluded from this study. It is up to future research to juxtapose these approaches and weigh their advantages and disadvantages. Furthermore, we rely on the full information maximum likelihood estimator which assumes that the observed variables follow a multivariate normal distribution^11^. In empirical research, this assumption can be too restrictive. For example, climate resilience indicators were found to violate the normality assumption^62^. Fortunately, most SEM implementations offer adjustments to the maximum likelihood estimator to make it robust against a violation of the normality assumption, e.g.,^63^. Similarly, most implementations offer estimators that can deal with ordinally scaled observed variables such as weighted means and variance adjusted weighted least squares [WLSMV, ^64,65^]. Ordinally scaled variables are not uncommon in ecological research, see, e.g.,^66^. Furthermore, we limit ourselves to modeling additive composites, i.e., composites that are linear combinations of other variables. However, there are also multiplicative composites such as the potential ecological risk index, which is defined as the product of the contamination factor and toxicological response factor of individual heavy metals^67^. Although multiplicative composites can be linearized using the logarithm, it is up to future research to provide ways to model this type of composites. Moreover, considering fixed-weight composites, we have only explained how the H–O specification can be used to model composites as sum or average of their components. Future research needs to show how the weights of the composites of interest can be fixed at unequal values. This would allow ecologists to analyze the sensitivity of the weight attribution for a composite when there is no solid scientific theory to support a particular set of weights^68^.

## Data Availability

The R code is accessible via the following URL: https://osf.io/c7jz2/?view_only=e4722965ce3b4ffbb0890a2d55868d93.

## References

[1] Grace, J. B., Anderson, T. M., Olff, H. & Scheiner, S. M. On the specification of structural equation models for ecological systems. *Ecol. Monogr.* **80**, 67–87 (2010).

[2] Pugesek, B. H. *Structural equation modeling: Applications in ecological and Evolutionary Biology* (Cambridge University Press, 2009).

[3] Arhonditsis, G. et al. Exploring ecological patterns with structural equation modeling and Bayesian analysis. *Ecol. Model.* **192**, 385–409 (2006).

[4] Malaeb, Z. A., Summers, J. K. & Pugesek, B. H. Using structural equation modeling to investigate relationships among ecological variables. *Environ. Ecol. Stat.* **7**, 93–111 (2000).

[5] Eisenhauer, N., Bowker, M. A., Grace, J. B. & Powell, J. R. From patterns to causal understanding: Structural equation modeling (SEM) in soil ecology. *Pedobiologia* **58**, 65–72 (2015).

[6] Zyphur, M. J., Bonner, C. V. & Tay, L. Structural equation modeling in organizational research: The state of our science and some proposals for its future. *Annu. Rev. Organ. Psych. Organ. Behav.* **10**, 495–517 (2023).

[7] MacCallum, R. C. & Austin, J. T. Applications of structural equation modeling in psychological research. *Annu. Rev. Psychol.* **51**, 201–226 (2000). 10751970 10.1146/annurev.psych.51.1.201

[8] Hoyle, R. H. Introduction to the special section: Structural equation modeling in clinical research. *J. Consult. Clin. Psychol.* **62**, 427–428 (1994). 8063969 10.1037//0022-006x.62.3.427

[9] Gefen, D., Rigdon, E. E. & Straub, D. An update and extension to SEM guidelines for administrative and social science research. *MIS Q.* **35**, iii–xiv (2011).

[10] Graham, J. M. The general linear model as structural equation modeling. *J. Educ. Behav. Stat.* **33**, 485–506 (2008).

[11] Bollen, K. A. *Structural equations with latent variables* (Wiley, 1989).

[12] Garrido, M., Hansen, S. K., Yaari, R. & Hawlena, H. A model selection approach to structural equation modelling: A critical evaluation and a road map for ecologists. *Methods Ecol. Evol.* **13**, 42–53 (2021).

[13] Frauendorf, M. et al. Conceptualizing and quantifying body condition using structural equation modelling: A user guide. *J. Anim. Ecol.* **90**, 2478–2496 (2021). 34437709 10.1111/1365-2656.13578 PMC9291099

[14] Grace, J. B. *Structural equation modeling and natural systems* (Cambridge University Press, 2006).

[15] Borsboom, D. Latent variable theory. *Measurement: Interdisciplinary Research & Perspective* **6**, 25–53 (2008).

[16] Liu, X. et al. Linking individual-level functional traits to tree growth in a subtropical forest. *Ecology* **97**, 2396–2405 (2016). 27859093 10.1002/ecy.1445

[17] Bollen, K. A. & Noble, M. D. Structural equation models and the quantification of behavior. *Proc. Natl. Acad. Sci. U.S.A.* **108**, 15639–15646 (2011). 21730136 10.1073/pnas.1010661108 PMC3176611

[18] Cubaynes, S. et al. Testing hypotheses in evolutionary ecology with imperfect detection: Capture-recapture structural equation modeling. *Ecology* **93**, 248–255 (2012). 22624306 10.1890/11-0258.1

[19] Jones, C. M. et al. Recently identified microbial guild mediates soil sink capacity. *Nat. Clim. Change* **4**, 801–805 (2014).

[20] Hopcraft, J. G. C., Anderson, T. M., Pérez-Vila, S., Mayemba, E. & Olff, H. Body size and the division of niche space: food and predation differentially shape the distribution of serengeti grazers. *J. Anim. Ecol.* **81**, 201–213 (2011). 21801174 10.1111/j.1365-2656.2011.01885.x

[21] Schuberth, F., Schamberger, T., Kemény, I. & Henseler, J. The sum score model: Specifying andtesting equally weighted composites using structural equation modeling. *Psychometrika* (forthcoming).

[22] Hu, A. et al. Ecological networks of dissolved organic matter and microorganisms under global change. *Nat. Commun.* **13**, 1–15 (2022). 35739132 10.1038/s41467-022-31251-1 PMC9226077

[23] Grace, J. B. & Bollen, K. A. Representing general theoretical concepts in structural equation models: The role of composite variables. *Environ. Ecol. Stat.* **15**, 191–213 (2008).

[24] Kline, R. B. *Principles and practice of structural equation modeling* 5th edn. (Guilford Press, 2023).

[25] Schuberth, F. The Henseler-Ogasawara specification of composites in structural equation modeling: A tutorial. *Psychol. Methods* **28**, 843–859 (2023). 34914475 10.1037/met0000432

[26] Dijkstra, T. K. & Henseler, J. Linear indices in nonlinear structural equation models: Best fitting proper indices and other composites. *Qual. Quant.* **45**, 1505–1518 (2011).

[27] Antoninka, A., Reich, P. B. & Johnson, N. C. Seven years of carbon dioxide enrichment, nitrogen fertilization and plant diversity influence arbuscular mycorrhizal fungi in a grassland ecosystem. *New Phytol.* **192**, 200–214 (2011). 21651560 10.1111/j.1469-8137.2011.03776.x

[28] Henseler, J. *Composite-based structural equation modeling: Analyzing latent and emergent variables* (Guilford Press, 2021).

[29] Eldridge, D. J., Delgado-Baquerizo, M., Travers, S. K., Val, J. & Oliver, I. Do grazing intensity and herbivore type affect soil health? Insights from a semi-arid productivity gradient. *J. Appl. Ecol.* **54**, 976–985 (2016).

[30] Verheyen, K., Guntenspergen, G. R., Biesbrouck, B. & Hermy, M. An integrated analysis of the effects of past land use on forest herb colonization at the landscape scale. *J. Ecol.* **91**, 731–742 (2003).

[31] Chaudhary, V. B. et al. Untangling the biological contributions to soil stability in semiarid shrublands. *Ecol. Appl.* **19**, 110–122 (2009). 19323176 10.1890/07-2076.1

[32] Gough, L. & Grace, J. B. Effects of environmental change on plant species density: Comparing predictions with experiments. *Ecology* **80**, 882–890 (1999).

[33] Laughlin, D. C. & Abella, S. R. Abiotic and biotic factors explain independent gradients of plant community composition in ponderosa pine forests. *Ecol. Model.* **205**, 231–240 (2007).

[34] Grace, J. B. Structural equation modeling for observational studies. *J. Wildl. Manag.* **72**, 14–22 (2008).

[35] Hu, A. et al. Mountain biodiversity and ecosystem functions: Interplay between geology and contemporary environments. *ISME J.* **14**, 931–944 (2020). 31896789 10.1038/s41396-019-0574-x PMC7082341

[36] Allison, P. D. Missing data techniques for structural equation modeling. *J. Abnorm. Psychol.* **112**, 545–557 (2003). 14674868 10.1037/0021-843X.112.4.545

[37] MacCallum, R. C. & Browne, M. W. The use of causal indicators in covariance structure models: Some practical issues. *Psychol. Bull.* **114**, 533–541 (1993). 8272469 10.1037/0033-2909.114.3.533

[38] Ogasawara, H. Asymptotic expansions of the distributions of estimators in canonical correlation analysis under nonnormality. *J. Multivar. Anal.* **98**, 1726–1750 (2007).

[39] Yu, X., Schuberth, F. & Henseler, J. Specifying composites in structural equation modeling: A refinement of the Henseler-Ogasawara specification. *Stat. Anal. Data Min. ASA Data Sci. J.* **16**, 348–357 (2023).

[40] Henseler, J. & Schuberth, F. Using confirmatory composite analysis to assess emergent variables in business research. *J. Bus. Res.* **120**, 147–156 (2020).

[41] Henseler, J. & Schuberth, F. in *Auxiliary theories* (ed.Henseler, J.) *Composite-based Structural Equation Modeling: Analyzing Latent and Emergent Variables* Ch. 2, 25–37 (The Guilford Press, 2021).

[42] Dijkstra, T. K. A perfect match between a model and a mode. *Partial Least Squares Path Modeling* 55-80 (2017).

[43] Rosseel, Y. lavaan: An R package for structural equation modeling. *J. Stat. Softw.* **48**, 1–36 (2012).

[44] Muthén, L. K. & Muthén, B. O. *Mplus* 8th edn (Muthén & Muthén, Los Angeles, CA, 1998-2017).

[45] Arbuckle, J. L. *Amos 27.0 user’s guide* (IBM SPSS, 2020).

[46] Jöreskog, K. G. A general method for analysis of covariance structures. *Biometrika* **57**, 239–251 (1970).

[47] Browne, M. W. Generalized least squares estimators in the analysis of covariance structures. *S. Afr. Stat. J.* **8**, 1–24 (1974).

[48] Schamberger, T., Schuberth, F. & Henseler, J. Confirmatory composite analysis in human development research. *Int. J. Behav. Dev.* **47**, 89–100 (2022).

[49] Nunnally, J. C. & Bernstein, I. H. *Psychometric theory* (McGraw-Hill, 1994).

[50] Browne, M. W. & Cudeck, R. Alternative ways of assessing model fit. *Sociol. Methods Res.* **21**, 230–258 (1992).

[51] Hu, L. & Bentler, P. M. Cutoff criteria for fit indexes in covariance structure analysis: Conventional criteria versus new alternatives. *Struct. Equ. Model.* **6**, 1–55 (1999).

[52] Akaike, H. A new look at the statistical model identification. *IEEE Trans. Autom. Control* **19**, 716–723 (1974).

[53] Schwarz, G. Estimating the dimension of a model. *Ann. Stat.* **6**, 461–464 (1978).

[54] Sclove, S. L. Application of model-selection criteria to some problems in multivariate analysis. *Psychometrika* **52**, 333–343 (1987).

[55] Cohen, P., Cohen, J., Teresi, J., Marchi, M. & Velez, C. N. Problems in the measurement of latent variables in structural equations causal models. *Appl. Psychol. Meas.* **14**, 183–196 (1990).

[56] Schuberth, F., Schamberger, T. & Henseler, J. *More powerful parameter tests? No, rather biased parameter estimates* (Some reflections on path analysis with weighted composites, Behavior Research Methods, 2023). 10.3758/s13428-023-02256-5 PMC11133201 37936011

[57] Van Riel, A. C. R., Henseler, J., Kemény, I. & Sasovova, Z. Estimating hierarchical constructs using consistent partial least squares: The case of second order composites of common factors. *Ind. Manag. Data Syst.* **117**, 459–477 (2017).

[58] Dijkstra, T. K. & Henseler, J. Consistent and asymptotically normal PLS estimators for linear structural equations. *Computat. Stat. Data Anal.* **81**, 10–23 (2015).

[59] Hwang, H. et al. An approach to structural equation modeling with both factors and components: Integrated generalized structured component analysis. *Psychol. Methods* **26**, 273–294 (2021). 32673042 10.1037/met0000336

[60] Wold, H. in *Soft modeling: The basic design and some extensions* (eds Jöreskog, K. G. & Wold, H.) *Systems under Indirect Observation: Causality, Structure, Prediction Part II* 1–54 (North-Holland, 1982).

[61] Rose, N., Wagner, W., Mayer, A. & Nagengast, B. Model-based manifest and latent composite scores in structural equation models. *Collabra: Psychology* **5** (2019).

[62] Feldmeyer, D., Wilden, D., Jamshed, A. & Birkmann, J. Regional climate resilience index: A novel multimethod comparative approach for indicator development, empirical validation and implementation. *Ecol. Ind.* **119**, 106861 (2020).

[63] Satorra, A. & Bentler, P. M. Corrections to test statistics and standard errors in covariance structure analysis. In *Latent variable analysis: Applications for developmental research* (eds von Eye, A. & Clogg, C. C.) 399–419 (SAGE Publications, 1994).

[64] Muthén, B. A general structural equation model with dichotomous, ordered categorical, and continuous latent variable indicators. *Psychometrika* **49**, 115–132 (1984).

[65] Lee, S.-Y., Poon, W.-Y. & Bentler, P. Full maximum likelihood analysis of structural equation models with polytomous variables. *Stat. Prob. Lett.* **9**, 91–97 (1990).

[66] Podani, J. Multivariate exploratory analysis of ordinal data in ecology: Pitfalls, problems and solutions. *J. Veg. Sci.* **16**, 497–510 (2005).

[67] Keshavarzi, A. & Kumar, V. Spatial distribution and potential ecological risk assessment of heavy metals in agricultural soils of Northeastern Iran. *Geol. Ecol. Landscapes* **4**, 87–103 (2019).

[68] Van de Kerk, G. & Manuel, A. R. A comprehensive index for a sustainable society: The ssi - the sustainable society index. *Ecol. Econ.* **66**, 228–242 (2008).

